# Association of central α-klotho with dopaminergic and melanocortin receptors on feed intake regulation in chicken

**DOI:** 10.1016/j.psj.2026.107042

**Published:** 2026-05-01

**Authors:** Yaser Hassania, Shahin Hassanpour, Morteza Zendehdel, Bita Vazir

**Affiliations:** a Faculty of Veterinary Medicine, SR.C., Islamic Azad University, Tehran, Iran; b Department of Veterinary Basic Sciences, SR.C., Islamic Azad University, Tehran, Iran; c Department of Basic Sciences, Faculty of Veterinary Medicine, University of Tehran, 14155-6453 Tehran, Iran

**Keywords:** α-klotho, Dopaminergic, Melanocortin, Feed intake, Chicken

## Abstract

This study aimed to detemine association of central α-klotho with dopaminergic and melanocortin receptors on feed intake regulation in chicken. 264 meat-type chicken were included 6 experiments, each containing 4 groups. In experiment 1, the group (A) received injections of saline, group (B) SCH23390 (D_1_ receptor antagonist, 5 nmol), group (C) α-klotho (4 µg) and group (D) co-injection of the SCH23390 + α-klotho. In experiments 2-6, AMI-193 (D_2_ receptor antagonist, 5 nmol), NGB2904 (D_3_ receptor antagonist, 5 nmol), L-741,742 (D_4_ receptor antagonist, 5 nmol), SHU9119 (nonselective MC_3_/MC_4_ receptor antagonist, 0.5 nmol) and MCL0020 (selective MC_4_ receptor antagonist, 0.5 nmol), instead of the SCH23390. Then feed intake determined up to 120 minutes post injection. Based on the results, D_1_ receptor antagonist decreased α-klotho-induced hypophagic effect compared to control group (P < 0.05). Also, D_2_ receptor antagonist decreased α-klotho-induced hypophagic effect compared to control group (P < 0.05). Co-injection of the D_3_ receptor antagonist + α-klotho had no effect on α-klotho-induced hypophagia (P > 0.05). Co-injection of the D_4_ receptor antagonist + α-klotho had no effect on α-klotho-induced hypophagia (P > 0.05). Co-injection of the nonselective MC_3_/MC_4_ receptor antagonist decreased α-klotho-induced hypophagic effect compared to control group (P < 0.05). Co-injection of the selective MC_4_ receptor antagonist decreased α-klotho-induced hypophagic effect compared to control group (P < 0.05). Findings suggested α-klotho-induced hypophagia mediates by D_1_ and D_2_ dopaminergic as well as MC_3_ and MC_4_ melanocortin receptors in neonatal broiler chicken.

## Introduction

The energy condition of an organism is a crucial element that affects both its survival and growth. When there is an energy shortage, it is addressed by increasing food intake, a behavioral change managed by the brain's neural mechanisms (Abd El-Hack et al., 2022). The regulation of nutritional consumption is carefully governed by internal physiological cues and external environmental factors. The hypothalamus and brainstem process neural and hormonal data from peripheral systems and different areas of the brain to trigger feeding actions (Holt, 2022). The arcuate nucleus (ARC) located in the hypothalamus and the nucleus of the solitary tract (NTS) within the brainstem are key brain areas that receive serotonergic inputs from the dorsal raphe nucleus (DRN). The ARC is found near the third ventricle and has a leaky blood–brain barrier (BBB) (Holt, 2022). This particular anatomical arrangement allows it to efficiently process circulating nutritional and hormonal signals. Consequently, the ARC leverages this information to influence feeding behavior by sending projections to nearby hypothalamic nuclei and reward-related mesolimbic pathways. The NTS is vital for integrating peripheral visceral afferents, including vagal afferents, along with hormonal signals related to energy balance (Chen and Zhan, 2023). The two previously mentioned regions have been thoroughly researched regarding food intake, as they contain neuronal populations critical for sustaining nutritional balance: neurons that express POMC and those that produce agouti-related peptide (AgRP) and neuropeptide Y (NPY). Both POMC neurons in the ARC and those in the NTS exert their appetite-suppressing effects through the melanocortin signaling pathway (Brüning and Fenselau, 2023).

α-klotho is a protein linked to longevity, taking its name from the Greek goddess klotho, who is known for "spinning the thread of life" (Monteiro et al., 2016). Following its discovery, interest in α-klotho has steadily grown, driven by the idea that variants of α-klotho mutations could provide essential insights into the processes of human aging. This fascination has further increased, as changes in the human KL gene have been associated with differences in lifespan (Goel and Gude, 2014). α-klotho is crucial for maintaining brain health throughout human life, and its decline with age has been connected to neurodegenerative diseases and cognitive decline. Levels of α-klotho rise during early development but then begin to diminish with age in the cerebrospinal fluid. Moreover, studies indicate that the levels of this protein in the white matter of the brain significantly decline in older mouse and primate models (Hanson et al., 2021). α-klotho is crucial for the effective functioning of advanced cognitive abilities, and higher levels of soluble α-klotho found in serum correlate positively with cognitive performance. It has been suggested that brain-derived α-klotho could affect the functionality of neurons and oligodendrocytes in laboratory settings; however, in vivo studies related to cognitive functions such as learning and memory demonstrate that α-klotho is essential for maintaining the structural integrity of various tissues (Ge et al., 2024). α-klotho acts as a negative regulator for neurons that express NPY/AgRP in the arcuate nucleus (Landry et al., 2020). It has been observed that administering intraperitoneal α-klotho leads to a decrease in fat tissue accumulation and an increase in energy expenditure, without affecting food intake in mouse models (Rao et al., 2019). While peripheral α-klotho is involved in regulating metabolic functions, its large molecular size prevents it from crossing the blood-brain barrier (Nakajima et al., 2016). The role of α-klotho has recently been investigated in birds. Eslam-aghdam et al. (2024) showed that α-klotho-induced reduced feed intake occurs through NPY_1_ receptors in broiler chickens.

Dopamine (DA) is a neurotransmitter that plays a vital role in triggering behaviors linked to reward, arousal, cognition, and movement. Additionally, DA is significant in regulating feeding and feelings of fullness (Baik, 2021). Dopamine interacts with various G-protein-coupled receptors (GPCRs) D_1_-D_5_ and their variants. The central neural pathways involved in dopaminergic signaling are intricate, prominently featuring the nigrostriatal, mesolimbic/mesocortical, and tuberoinfundibular DA systems (Wallace and Fordahl, 2022). The influence of hypothalamic DA on food consumption is determined by its impact on specific hypothalamic nuclei, the participation of particular dopamine receptors, the overall metabolic condition, and the method of delivery for either food-related or pharmacological DA-modulating signals. Generally, DA functioning in two areas, the lateral hypothalamic area (LHA) and the ventromedial hypothalamus (VMH), seems to have opposing effects on food intake (Joshi et al., 2022).

The melanocortin system consists of five receptor subtypes, categorized as GPCRs, and has shown involvement in numerous biological pathways (Ericson et al., 2017). Numerous pieces of evidence indicate that the central melanocortin system is essential for controlling food consumption and energy output in avian species and rodents, particularly through MC_3_/MC_4_ receptors. Reports suggest that the ICV administration of α-MSH and β-MSH led to an anorexigenic effect in newborn chicks (Cao et al., 2020). Additionally, it has been reported that melanotan-II, a nonselective MC_3_/MC_4_ receptors agonist, can decrease food consumption in mice after ICV administration (Rowland et al., 2010).

Based on literature, there is report regarding interaction of the central α-klotho with dopaminergic and melanocortin receptors. Mice lacking α-klotho exhibit neurodegeneration of dopaminergic neurons in the substantia nigra and ventral tegmentum, whereas increased klotho expression safeguards dopaminergic neurons from oxidative damage. Exogenous α-klotho treatment shows neuroprotective effects in toxin rat models of Parkinson’s disease by reducing astrogliosis, apoptosis, and oxidative stress (Luthra et al., 2022). Thus, This study aimed to detemine association of central α-klotho with dopaminergic and melanocortin receptors on feed intake regulation in chicken.

## Material and methods

### Drugs

α-klotho (Tocris-CAS Number: 2102414-23-1.), SHU9119 (nonselective MC_3_/MC_4_ receptor antagonist, Tocris-CAS Number: 168482-23-3), MCL0020 (selective MC_4_ receptor antagonist, Tocris- CAS Number: 475498-26-1), SCH23390 (D_1_ receptor antagonist, Tocris-CAS Number: 125941-87-9), AMI-193 (D_2_ receptor antagonist, Tocris-CAS Number: 510-74-7), NGB2904 (D_3_ receptor antagonist, Tocris-CAS Number: 189061-11-8), L-741,742 (D_4_ receptor antagonist, Tocris-CAS Number: 874882-93-6) and Evan’s blue (Sigma-CAS Number: 314-13-6) were provided.

### Birds and grouping

264 meat-type (ROSS 308) day old chicken obtained from a domestic hatchery. The chicks were kept in groups for 2 days, then placed in solitary confinement until day 5. Birds had ad libitum access to water and starter diet (Table 1). This research included 6 experiments, each containing 4 groups (n = 11 repetitions in each group). In experiment 1, the group (A) received injections of saline, group (B) SCH23390 (5 nmol), group (C) α-klotho (4 µg) and group (D) co-injection of the SCH23390 + α-klotho. In experiment 2, the chicken in group (A) received an ICV injection of saline, group (B) AMI-193 (5 nmol), group (C) α-klotho (4 µg) and group (D) co-injection of the AMI-193 + α-klotho. In experiment 3, the chicken in group (A) received an ICV injection of saline, group (B) NGB2904 (5 nmol), group (C) α-klotho (4 µg) and group (D) co-injection of the NGB2904 + α-klotho. In experiment 4, the injections comprised group (A) saline solution, group (B) L-741,742 (5 nmol), group (C) α-klotho (4 µg) and group (D) co-injection of the L-741,742 + α-klotho. In experiment 5, the chicken in group (A) received an ICV injection of saline, group (B) SHU9119 (0.5 nmol), group (C) α-klotho (4 µg) and group (D) co-injection of the SHU9119 + α-klotho. In experiment 6, the chicken in group (A) received an ICV injection of saline, group (B) MCL0020 (0.5 nmol), group (C) α-klotho (4 µg) and group (D) co-injection of the MCL0020 + α-klotho.

**Table 1:** Ingredient and nutrient analysis of experimental diet.

Ingredient (%)		Nutrient analysis	
Corn	52.85	ME (kcal/g)	2850
Soybean meal, 48% CP	31.57	Crude protein (%)	21
Wheat	5	Linoleic acid (%)	1.69
Gluten meal, 61% CP	2.50	Crude fiber (%)	3.55
Wheat bran	2.47	Calcium (%)	1
Di-calcium phosphate	1.92	Available phosphorus (%)	0.5
Oyster shell	1.23	Sodium (%)	0.15
Soybean oil	1.00	Potassium (%)	0.96
Mineral premix	0.25	Chlorine (%)	0.17
Vitamin premix	0.25	Choline (%)	1.30
Sodium bicarbonate	0.21	Arginine (%)	1.14
Sodium chloride	0.20	Isoleucine (%)	0.73
Acidifier	0.15	Lysine (%)	1.21
DL-Methionine	0.10	Methionine (%)	0.49
Toxin binder	0.10	Methionine+Cystine (%)	0.83
L-Lysine HCl	0.05	Threonine (%)	0.70
Vitamin D3	0.1	Tryptophan (%)	0.20
Multi enzyme	0.05	Valine (%)	0.78

### ICV injection procedure

On the fifth day, an intracerebroventricular injection was carried out. The top of the alert chick is held in place by an acrylic device. A stencil with an opening was made and placed on the skull above the right ventricular area (Jonaidi et al., 2012). The Hamilton syringe needle was pushed 4 mm into the skull. Injections of 10 µL were carried out without applying any pressure (Saito et al., 2005). At the end of each trial, the chicks were euthanized via an intraperitoneal overdose (50 mg/kg) of sodium thiopental (Rotexmedica, Germany; following AVMA Guidelines for the Euthanasia of Animals 'No: S5.2.1.1′, Acceptable Methods; non-inhaled agents). The accuracy of the injection into the right ventricle was evaluated by decapitation at the end of the experiment and confirmed by the detection of Evans Blue in the injected area (Furuse et al., 1999).

### Feed intake measurment

Birds were FD_3_ prior to the study and were returned to their cage following the injection. Subsequently, the quantity of feed consumed was documented at 30, 60, and 120-minute intervals following the injection (Jonaidi et al., 2012). As each experiment was focused on interaction with specific receptor, the experiments are separate from each other and there was no need to compare or analysis with each other.

### Statistical analysis

Feed consumption was evaluated by calculating the proportion of body weight and assessed through repeated measures analysis of variance (ANOVA), with results displayed as mean ± SEM. The Tukey-Kramer test was employed to compare the means (P < 0.05).

## Results

**Fig. 1:**
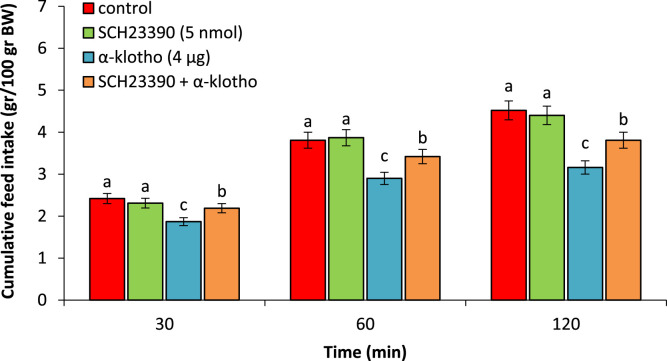
Effect of ICV injection of SCH23390 (5 nmol), α-klotho (4 µg) and their combination on cumulative food intake in neonatal chicken (n = 44). SCH23390: D1 receptor antagonist. Data are expressed as mean ± SEM. Different letters (a, b and c) indicate significant differences between treatments (P < 0.05).

In experiment 1, administering SCH23390 did not affect feed intake (P > 0.05). α-klotho (4 µg) caused a notable decrease in feed consumption when compared to the control group (P < 0.05). The concurrent administration of SCH23390 + α-klotho decreased hypophagic effects of the α-klotho (P < 0.05) (Fig. 1).

**Fig. 2:**
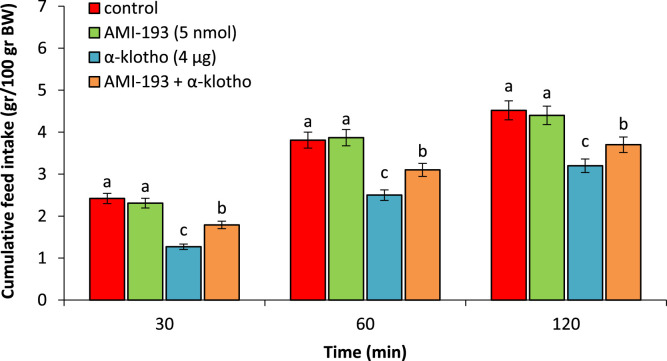
Effect of ICV injection of AMI-193 (5 nmol), α-klotho (4 µg) and their combination on cumulative food intake in neonatal chicken (n = 44). AMI-193: D2 receptor antagonist. Data are expressed as mean ± SEM. Different letters (a, b and c) indicate significant differences between treatments (P < 0.05).

According to experiment 2, administering AMI-193 (5 nmol) did not affect feed intake (P > 0.05). α-klotho (4 µg) caused a notable decrease in feed consumption when compared to the control group (P < 0.05). The concurrent administration of AMI-193 + α-klotho decreased hypophagic effects of the α-klotho (P < 0.05) (Fig. 2).

**Fig. 3:**
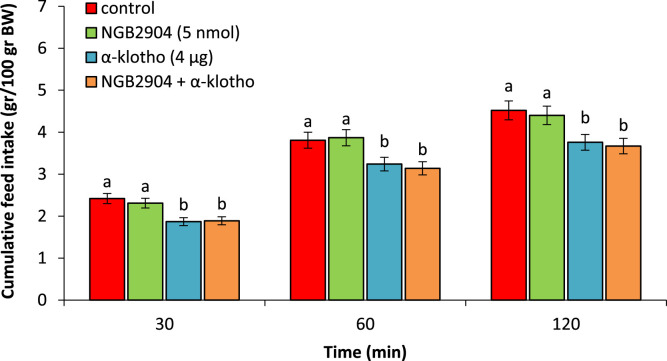
Effect of ICV injection of NGB2904 (5 nmol), α-klotho (4 µg) and their combination on cumulative food intake in neonatal chicken (n = 44). NGB2904: D3 receptor antagonist. Data are expressed as mean ± SEM. Different letters (a and b) indicate significant differences between treatments (P < 0.05).

Based on Fig. 3, ICV injection of the NGB2904 (5 nmol) had no significant effect on feed intake compared to control group (P > 0.05). α-klotho (4 µg) caused a notable decrease in feed consumption compared to the control group (P < 0.05). α-klotho-induced hypophagia was no affected by co-injection of the NGB2904 + α-klotho (P > 0.05) (Fig. 3).

**Fig. 4:**
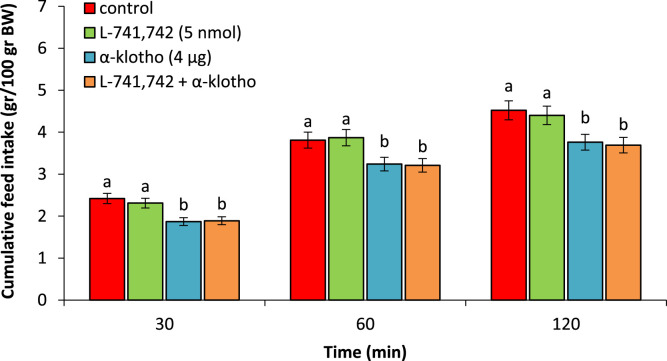
Effect of ICV injection of L-741,742 (5 nmol), α-klotho (4 µg) and their combination on cumulative food intake in neonatal chicken (n = 44). L-741,742: D4 receptor antagonist. Data are expressed as mean ± SEM. Different letters (a and b) indicate significant differences between treatments (P < 0.05).

As seen in Fig. 4, L-741,742 (5 nmol), had no significant effect on feed intake compared to control group (P > 0.05). α-klotho (4 µg) significantly lead to hypophagia compared to the control group (P < 0.05). Co-injection of the L-741,742 + α-klotho had no effect on α-klotho-induced (P > 0.05) (Fig. 4).

**Fig. 5:**
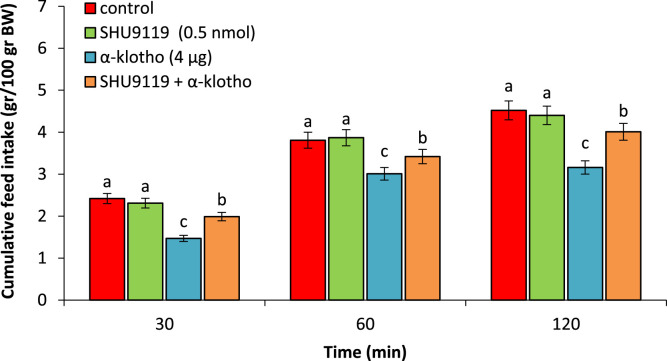
Effect of ICV injection of SHU9119 (0.5 nmol), α-klotho (4 µg) and their combination on cumulative food intake in neonatal chicken (n = 44). SHU9119: nonselective MC3/MC4 receptor antagonist. Data are expressed as mean ± SEM. Different letters (a, b and c) indicate significant differences between treatments (P < 0.05).

As observed, SHU9119 (0.5 nmol), had no significant effect on feed intake compared to control group (P > 0.05). α-klotho (4 µg) significant lead to hypophagia compared to the control group (P < 0.05). Co-injection of the SHU9119 + α-klotho decreased hypophagic effects of the α-klotho (P < 0.05) (Fig. 5).

**Fig. 6:**
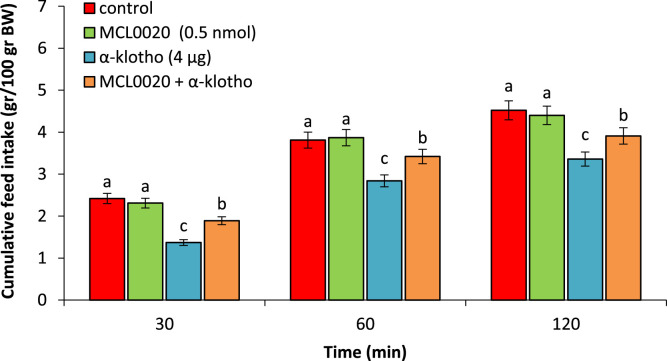
Effect of ICV injection of MCL0020 (0.5 nmol), α-klotho (4 µg) and their combination on cumulative food intake in neonatal chicken (n = 44). MCL0020: selective MC4 receptor antagonist. Data are expressed as mean ± SEM. Different letters (a, b and c) indicate significant differences between treatments (P < 0.05).

Also, MCL0020 (0.5 nmol), had no significant effect on feed intake compared to control group (P > 0.05). α-klotho (4 µg) significantly lead to hypophagia compared to the control group (P < 0.05). Co-injection of the MCL0020 + α-klotho decreased hypophagic effects of the α-klotho (P < 0.05) (Fig. 6).

## Discussion

The findings indicate that α-klotho diminished feed consumption. In one study, α-klotho has been investigated in birds. Eslam-aghdam et al. (2024) demonstrated that α-klotho-induced hypophagia occurs via NPY_1_ receptors in broiler chickens, and our findings supported this conclusion. Mechanistically, α-klotho functions as a scaffolding protein to enhance FGFR activity and subsequent PI3kinase signaling, a process deemed crucial for central α-klotho-mediated control of energy balance and NPY/AgRP neurons. ICV α-klotho treatment enhances the accumulation of liver fat, while central α-klotho inhibition swiftly disrupts glucose metabolism (Landry et al., 2020). All reported hypothalamic POMC neurons have shown a response to α-klotho. The signaling of PI3 kinase is essential for α-klotho's regulation of NPY/AgRP and POMC neurons (Landry et al., 2020). CSF concentrations of α-klotho diminish in neurological conditions, highlighting its importance in regulating energy use and hunger. Administering α-klotho into the ICV enhances liver lipid buildup, while central inhibition of α-klotho swiftly disrupts glucose metabolism (Landry et al., 2020).

Melanocortin system within the CNS governs various physiological functions such as food intake and energy balance regulation (Singhal and Hill, 2018). Only two of them MC_3_ and MC_4_ play a role in controlling central food intake (Micioni Di Bonaventura et al., 2020). Previous studies have indicated that after ICV administration of MC_3_ and MC_4_ receptor agonists and antagonists, feeding behaviors were both diminished and increased, respectively (Ahmadi et al., 2019; Micioni Di Bonaventura et al., 2020). Melanocortin receptors are found in the ARC and nucleus tractus solitarius where POMC is expressed. There exists a potential for neuroendocrine interactions between the central melanocortin system and the hormones that regulate food consumption and energy expenditure. For instance, leptin acts as a peptide exhibiting anorexigenic characteristics, resulting in reduced food consumption at the ARC by activating melanocortin receptors. POMC-expressing neurons in the ARC function as essential elements of the melanocortin system. These neurons display responsiveness to circulating substances and combine various excitatory and inhibitory inputs from different areas of the brain. They consequently manage various aspects of feeding behavior (Han et al., 2022).

Mice without α-klotho show neurodegeneration of dopaminergic neurons in the ventral tegmentum and substantia nigra, while enhanced klotho expression protects dopaminergic neurons from oxidative harm (Luthra et al., 2022). α-klotho over expression protects dopaminergic neurons against oxidative damage. Klotho down-regulation is associated with aging-related inflammation and the development of early-stage chronic kidney disease (Baluchnejadmojarad et al., 2017). ICV injection of α-klotho reduced apomorphine-induced rotations, clearly and indirectly indicating its ability to avert a decrease in striatal dopamine. Additionally, rats with 6-OHDA lesions demonstrated significant motor deficits in the narrow beam task, evidenced by increased latency to initiate the task and longer duration needed to cross the beam (Baluchnejadmojarad et al., 2017). This unusual feature in narrow beam in 6-OHDA-lesioned rats results from decreased dopamine levels in the affected striatum, leading to bradykinesia. The administration of α-klotho alleviated motor deficits because of its capacity to protect dopaminergic neurons from damage (Zhang et al., 2017). This indicates the significant role of PKA and CaMKII signaling in facilitating the protective effect of klotho (Kiasalari et al., 2016). it has been demonstrated that the activity of cAMP-dependent PKA is crucial for protecting dopaminergic neurons from oxidative stress induced by 6-OHDA, and its inhibition by H-89 results in cellular toxicity. Furthermore, it has asserted that the CaMKII cascade is essential for providing a neuroprotective effect (Zhang et al., 2017).

In summary, new discoveries for the first time demonstrated that α-klotho-induced hypophagia is mediated by D_1_ and D_2_ dopaminergic receptors along with MC_3_ and MC_4_ melanocortin receptors in neonatal broiler chickens. Nevertheless, there are no reports regarding their linkage to feed consumption in both mammals and birds. Recent discoveries are unique and can serve as foundational knowledge while justifying further studies required to uncover cellular and molecular functional mechanisms.

## CRediT authorship contribution statement

**Yaser Hassania:** Writing – original draft, Data curation. **Shahin Hassanpour:** Writing – review & editing, Supervision, Methodology. **Morteza Zendehdel:** Supervision, Methodology. **Bita Vazir:** Project administration.

## Disclosures

No conflict of interest.
